# Response to Adamson et al. (2020): ‘Cognitive behavioural therapy for chronic fatigue and chronic fatigue syndrome: Outcomes from a specialist clinic in the UK’

**DOI:** 10.1177/13591053211008203

**Published:** 2021-04-10

**Authors:** Brian M Hughes, David Tuller

**Affiliations:** 1 National University of Ireland, Galway, Galway, Ireland; 2 University of California, Berkeley, CA, USA

**Keywords:** chronic fatigue syndrome, cognitive behaviour therapy, health care systems, methodology, quantitative methods

## Abstract

In a paper published in the *Journal of the Royal Society of Medicine*, Adamson et al. (2020) interpret data as showing that cognitive behavioural therapy leads to improvement in patients with chronic fatigue syndrome and chronic fatigue. Their research is undermined by several methodological limitations, including: (a) sampling ambiguity; (b) weak measurement; (c) survivor bias; (d) missing data and (e) lack of a control group. Unacknowledged sample attrition renders statements in the published Abstract misleading with regard to points of fact. That the paper was approved by peer reviewers and editors illustrates how non-rigorous editorial processes contribute to systematic publication bias.

In a paper by Adamson et al. (2020), published in the October issue of the *Journal of the Royal Society of Medicine*, the authors interpret their data as revealing significant improvements following cognitive behavioural therapy (CBT) in a large sample of patients with chronic fatigue syndrome (CFS) and chronic fatigue (CF). In our view, their conclusions are misplaced and unwarranted. The paper and the research it describes are both problematic in several critical respects. For example, the Abstract – the section of the paper most likely to be read by clinicians – contains a crucial error in the way the data are described, and requires urgent correction.

In this review, we briefly survey the most compelling issues, and reflect on what the publication of such research implies about the nature and effectiveness of peer review in clinical behavioural science.

## Prelude: A conspicuous controversy overlooked

In explaining the rationale for the CFS-specific version of CBT used in their study, Adamson et al. (2020) write that the intervention is ‘based on a model which assumes that certain triggers such as a virus and/or stress trigger symptoms of fatigue. Subsequently symptoms are perpetuated inadvertently by unhelpful cognitive and behavioural responses’ (p. 396). Treatment involves, among other elements, ‘addressing unhelpful beliefs which may be interfering with helpful changes’ (p. 396).

This theory is essentially the one laid out more than 30 years ago, in a 1989 paper by a team that also included two of the current paper’s authors; namely, professors Wessely et al. (1989). The main problem here is that, in light of recent re-evaluations of key research in the field as well as accelerating efforts to unlock underlying pathophysiological processes, this cognitive-behavioural theory of CFS is now very widely disputed.

The authors’ failure to acknowledge that their decades-old theory is currently embroiled in a highly contentious academic dispute represents an omission that verges on selective reporting of the research history. Since 1989, a considerable volume of empirical research has documented a wide range of organic dysfunctions in patients. In 2015, a report from the US Institute of Medicine (now the National Academy of Medicine) cited this body of research when describing the illness as ‘a serious, chronic, complex, and multisystem disease’ (Institute of Medicine, 2015: 209) and rebutting claims that it is psychiatric or psychological in nature. By not mentioning research that would appear to counter their narrative, Adamson et al. fail to anticipate – or to attempt to offset – some obvious criticisms that their CBT-focused approach is likely to attract.

Adamson et al. were similarly selective in their brief discussion of the literature on interventions. A thorough review of this research would almost certainly have culminated in, at best, a far more lukewarm conclusion regarding the potential utility of CBT as a treatment for CFS and related conditions. In late 2020 (admittedly, some months after the authors submitted their paper to the *Journal of the Royal Society of Medicine*), the UK’s National Institute for Health and Care Excellence (NICE) published its own assessment of this literature. Having scrutinised in detail studies covering 172 CBT outcomes – findings previously used to support claims that CBT is an effective treatment for CFS and related conditions – NICE classified *all* of the research as constituting evidence of either ‘low’ or ‘very low’ quality. Across the entire literature, as judged by NICE, not a single claim for CBT efficacy was supported by any evidence that exceeded the ‘low quality’ threshold (NICE, 2020).

However, the shortcomings of the new paper by Adamson et al. extend far beyond a questionable theoretical premise or a selective literature review. Overall, the research is hampered by several fundamental methodological limitations that are not acknowledged sufficiently, or at all, by the authors. These include: (a) sampling ambiguity; (b) weak measurement; (c) survivor bias; (d) missing data and (e) lack of a control group. Given these issues, in our view, the findings reported by Adamson et al. are unreliable because they are very seriously inflated.

## Sampling ambiguity: Who were the participants?

The investigators seem confused about whether they are investigating patients with CF or patients with CFS. The title suggests the answer is both, but the paper itself generally refers to CFS throughout and to the participants as having met CFS criteria.

All 995 participants met the criteria outlined in the 2007 NICE guidance for what it called CFS/ME. These criteria require 4 months of fatigue. Yet, according to Adamson et al., only 76% met the Oxford case definition, which requires 6 months of fatigue and no other symptoms, and just 52% met the CDC criteria, which require 6 months of fatigue plus four of eight other symptoms. This raises a question as to whether 24% of the present sample had fatigue only for between 4 and 6 months. That seems hard to understand, given that participants were reported to have been ill for a mean duration of 6.64 years.

Nor is it clear if many or any of the included participants experienced post-exertional malaise, widely acknowledged as being a core symptom of the disease (e.g. CDC, 2021). Without more information, it is difficult to determine how many people in this study had CFS per se, as opposed to idiopathic CF or another illness in which fatigue was a symptom.

## Weak measurement: How valid were the outcomes?

The course of CBT described by Adamson et al. included up to 20 sessions on a twice-monthly basis. Patients completed several questionnaires at the start of treatment, at the fourth and seventh sessions, at discharge, and at 3 months after discharge. The measures included the SF-36 and the Chalder Fatigue Questionnaire (CFQ), along with more generic scales, such as those for work and social adjustment, depression and anxiety and overall health.

It is important to note that all of these measures are subjective. The study included no objective indicators often used in research assessing outcomes in patients being treated for disabling conditions. For example, the authors report no data on improvements in physical endurance (such as a walk test), fitness (such as a step test) or occupational well-being (such as return-to-work rates or changes in disability-related benefit payments). We can note that in past intervention research with CFS, CBT-based therapies that were reported as having led to self-reported ‘improvements’ were found to have had no effect whatsoever on either physical endurance, fitness or socio-economic outcome (Stouten, 2017).

Moreover, as this study was not blinded, all participants knew that they were receiving an intervention that was designed to help them. It should therefore not be surprising that some people receiving such an intervention would self-report short-term ephemeral benefits, in line with their expectations. Without any objective outcome measures, the risk of confirmation bias in such a study design is extremely high.

## Survivor bias: How meaningful were the results?

For several reasons, the main results do not support the interpretation that treatment was effective. Scores on the SF-36 rose from a mean of 47.6 at baseline to 57.5 at discharge and 58.5 at 3-month follow-up. In previous research on CFS, SF-36 scores of 65 or below have been used to identify serious disability and thus have been employed as inclusion criteria to determine whether participants are sick enough to be recruited for a treatment study. Notably, the present paper includes authors who have previously used the SF-36 in precisely this way (White et al., 2007). Therefore, these authors should be well aware that any treatment outcome in which SF-36 scores average 58.5 needs to be considered against the fact that patients overall remain seriously disabled despite undergoing therapy. CFQ scores at discharge and follow-up tell a similar story: while modestly improved from baseline, they nonetheless represent disablingly high levels of fatigue.

But even these results are likely to be misleading given the significant rate at which participants dropped out of treatment. Of the sample of 995 participants initially identified, some 31% were considered ‘lost-to-follow-up’ – as defined by the investigators, that meant they provided no data either at the end of treatment or at the follow-up assessment 3 months later, despite providing some data at the earlier timepoints. Moreover, their attrition was non-random: those who were lost-to-follow-up had reported, at baseline, greater problems with depression, work and social adjustment and physical function than those whose data were ultimately analysed.

Simply put, we have no idea what happened to almost a third of the participants, although we know that they were in relatively poor shape to begin with. Perhaps they were lost-to-follow-up because of further deteriorations in health, whether or not these were related to CBT, or perhaps because they just found CBT to be unhelpful.

The substantial attrition rate suggests an obvious problem of survivor bias. Any positive findings accruing from the whittled down dataset may simply be the inflationary result of a statistical artefact. Deep in the body of the text, the authors allude to this problem, stating that ‘there may have been some bias in the data, in that those who completed treatment may not represent all patients’ (p. 401). This modest acknowledgement falls short of the appropriate scientific rigour. It would have been more accurate to have stated that ‘an unknown amount of bias in these data is inevitable, in that those who completed treatment will not represent all patients’.

Adamson et al. make no mention of participants being lost-to-follow-up when summarising the findings in their Abstract. Instead, they state that data were available ‘for 995 patients’ before then stating that ‘85% of patients’ self-reported improvement after therapy. Their construction is extremely misleading. A crucial caveat – that the ‘85%’ applies only to a non-random subset of participants who were not lost-to-follow-up – is omitted. The resulting abstract presents an opaque sequence of points that serves to greatly inflate the findings, and which constitutes factual error.

Also in the Abstract, the authors highlight that ‘90%’ of patients ‘were satisfied with their treatment’. Again, that impressive-looking figure does not include responses from the 31% who were lost-to-follow-up. As the denominator is not explained, the Abstract’s description of this high approval rate is difficult even to understand.

## Missing data: What should we make of non-responses?

In addition to those participants who were classified as lost-to-follow-up, a further problem arises from the fact that, for several key variables, large numbers of the participants who remained did not complete the required questionnaires or return relevant data. Only 581 participants (58% of the initial sample of 995) completed the CFQ at the end of treatment and only 503 (51%) did so at follow-up. Only 441 participants (44%) completed the SF-36 at discharge, and just 404 (41%) did so at follow-up. Despite this, both CFQ and SF-36 scores are used as measures of treatment outcomes. Once again, when citing these results in their Abstract, the authors do not mention that data were missing for up to 6 out of every 10 participants.

In short, it is misleading for the authors to have set out positive findings without revealing that, for key outcome variables, conclusions were drawn from a substantially depleted dataset. This is especially true when participant disengagement *en masse* will almost certainly be suggestive of widespread treatment failure.

## Lack of a control group: What was the point?

It is an elementary principle of good study design that causality cannot be established without reference to a control group or control condition. The present study did not include a control group or control condition. Therefore, the study data cannot be used to support inferences about causality.

Nonetheless, in their discussion section, Adamson et al. write the following: ‘The cognitive behavioural therapy intervention *led to* significant improvements in patients’ self-reported fatigue, physical functioning and social adjustment’ (p. 400; our emphasis). This is a straightforward statement of causality and so is clearly unwarranted; in the absence of a control group, any such inference is unsound.

When further discussing their conclusions, the authors then state: ‘the lack of a control condition limits us from drawing any causal inferences, as we cannot be certain that the improvements seen are due to cognitive behavioural therapy alone and not any other extraneous variables’ (p. 401). Despite its implication of rigour, this statement includes another assertion of causality. Moreover, it is self-contradictory. To state that improvements might not be ‘due to CBT alone’ is to posit, as fact, that they are due to CBT at least in part but that other factors might have contributed. In one sentence, therefore, the authors draw a causal inference while denying the possibility of being able to do just that given their study design.

The paper by Adamson et al. does not present evidence that CBT ‘led to’ anything. The authors have provided a *partial* dataset suggesting that *some* of their participants self-reported *modest* increases in *subjective* assessments of well-being (while nonetheless remaining within a range of scores that indicate *severe debilitation*). These changes in scores might well have happened whether or not CBT had been administered.

## Conclusion: Therapeutic loyalties and the challenge of scientific reviewing

In our view, the shortcomings in the paper by Adamson et al. are as obvious as they are inherent. In that regard, given that it was approved by multiple peer reviewers and editors, we feel we must reflect on what its publication can teach us about the nature of contemporary scientific publication practices.

Even the most objective scientists will be strained by the problems of confirmation bias, especially if they have invested their professional reputations in a particular approach to therapy. This problem, sometime referred to as ‘therapeutic allegiance’, has been shown to present a statistically significant source of investigator bias in psychotherapy trials. In short, researchers are inclined to report larger effect sizes in studies of therapies about which they hold strong professional beliefs (Dragioti et al., 2015). It is undoubtedly the case that the authors of the Adamson et al. paper include some who have published widely on the use of CBT for CFS and related conditions, and who have advocated for its use over several decades. Based on the findings of numerous empirical studies of this class of bias, we join with other scholars who have called for therapeutic allegiance to be recognised as an important risk to research integrity, the details of which should always be publicly acknowledged, akin to those of any other conflict of interest.

Editors and peer reviewers vary in their theoretical backgrounds, areas of technical expertise and editorial philosophies. The peer review system relies significantly on volunteer effort, and this feature alone is one that affords it considerable esteem. The system, and those who participate in it, are deserving of our respect. The *Journal of the Royal Society of Medicine* operates a policy of publishing the names of peer reviewers for every article. Presumably, this is intended to support transparency, by allowing readers to evaluate for themselves the nature and scope of peer review in each given case.

The names of four peer reviewers were published for the paper by Adamson et al. These comprised, in turn: (a) a general practitioner and psychotherapist who specialises in ‘whole person’ psychotherapy; (b) a CBT therapist, whose personal website describes them as a ‘philanthropist’ and ‘entrepreneur’; (c) a biostatistician who serves as ‘Statistical Advisor’ to the *Journal of the Royal Society of Medicine* and (d) a junior doctor and psychiatry trainee.

Based on the Google Scholar database, the first and second peer reviewers have each published a small number of scholarly papers; both have a *h*-index of 2. The third peer reviewer has an extensive record of academic publication, which includes a paper co-authored with a prominent CFS researcher who himself has published extensively with two of the co-authors on the Adamson et al. paper (namely Wessely and Chalder). The fourth peer reviewer has co-authored one published paper. Overall, in our view, it is simply unclear whether the peer reviewers in this case had detailed experience with quantitative behavioural research or specialist knowledge in associated methodological issues, such as familiarity with the particular pitfalls arising from subtle demand characteristics that so often undermine research validity in behavioural medicine contexts.

More notably, the fourth peer reviewer is a psychiatry trainee on the Maudsley Training Programme. This programme is operated in partnership with the Institute of Psychiatry, Psychology and Neuroscience at King’s College London and the South London and Maudsley NHS Foundation Trust. All five co-authors of the Adamson et al. paper list one or both of these institutions as their academic affiliation(s). Two of the co-authors (Wessely, Chalder) hold senior positions at the Institute of Psychiatry at King’s. Another co-author (Santhouse) serves as a College Tutor and Clinical Skills Programme Co-ordinator on the Maudsley Training Programme itself. Ordinarily, being affiliated to the same institution(s) as a manuscript co-author would disqualify a person from serving as peer reviewer. Being affiliated not only to the same institution, but to the same academic units as *all* five co-authors would certainly appear to present significant issues concerning potential conflicts of interest. That the peer reviewer in question is a trainee of the co-authors’ institutions further compounds the problem. In our view, to ask a student to serve as peer reviewer for a paper produced by senior professors of their own college places them in an impossible position, and is highly inappropriate.

Aside from the peer review process, another conflict-of-issue arises. In intertwined professions such as academia and medicine, it can be extremely difficult to ensure or maintain author anonymity during the review process, or to completely obliterate the risk that conflicts of interest might skew the evaluations of reviewers (Hughes, 2018). Therefore, every effort must be made to avoid even the appearance of such a conflict. The *Journal of the Royal Society of Medicine* maintains editorial separation from the Royal Society of Medicine, a laudable principle of which it can be proud. That said, the fact that the Adamson et al. paper lists among its authors the outgoing president of the Royal Society of Medicine (i.e. Wessely) will likely confuse those readers who are unfamiliar with, or distrusting of, such safeguards. It is always preferable that, where possible, research authors submit their manuscripts to journals with which they have no association, even if tenuous. In our view, given widespread interest in the subject matter of the paper by Adamson et al., many such alternative options were available to these authors.

A critical problem concerns the factual inaccuracy of the Abstract of the Adamson et al. paper, which contains erroneous statements about the nature of the dataset that inflate the findings in a highly misleading fashion. As published, the Abstract implies that at least three separate statistical findings were based on a sample of 995 cases. In reality, because of both significant participant drop-out and widespread missing data, the study sample was actually hundreds of cases smaller in each instance. For example, stating that ‘Data were available for 995 patients’ and then that ‘85% of patients self-reported that they felt an improvement’ is factually incorrect, because around a third of the 995 patients had dropped out of the study and up to half of the self-report data was missing. Such sample attrition should be seen as a fatal shortcoming in any treatment study; the withdrawal of large numbers of cases distorts findings because statistical analyses end up disproportionately focussing on those patients for whom therapy was most beneficial. For it to have occurred on such a scale without being mentioned in the Abstract creates significant problems, and renders the resulting presentation of statements critically misleading with regard to points of fact. In our view, the error is substantive and requires the publication of a formal correction to Adamson et al.’s Abstract.

We agree with Adamson et al. that clinics should routinely assess treatment outcomes and report on change in naturalistic settings. We also very much agree with them that future studies should aim to employ better research methodologies. In our view, all such research should meet robust and rigorous standards of reliability and validity, and should be thoroughly evaluated against those standards. As such, we feel that the recently published paper by Adamson et al. is especially problematic: its methodology is hampered by a litany of grave shortcomings, and its conclusions presented with a level of confidence far exceeding what is warranted by its theoretical premise, dataset or study design.
